# Supplementary material for: Research on Extraction, Structure Characterization and Immunostimulatory Activity of Cell Wall Polysaccharides from Sparassis latifolia
Source: Polymers (Basel). 2022 Jan 28;14(3):549. doi: 10.3390/polym14030549 (PMC8840611; doi:10.3390/polym14030549)
Supplement: Supplementary file 1 [file polymers-14-00549-s001.zip › polymers-1454725-supplementary.pdf]

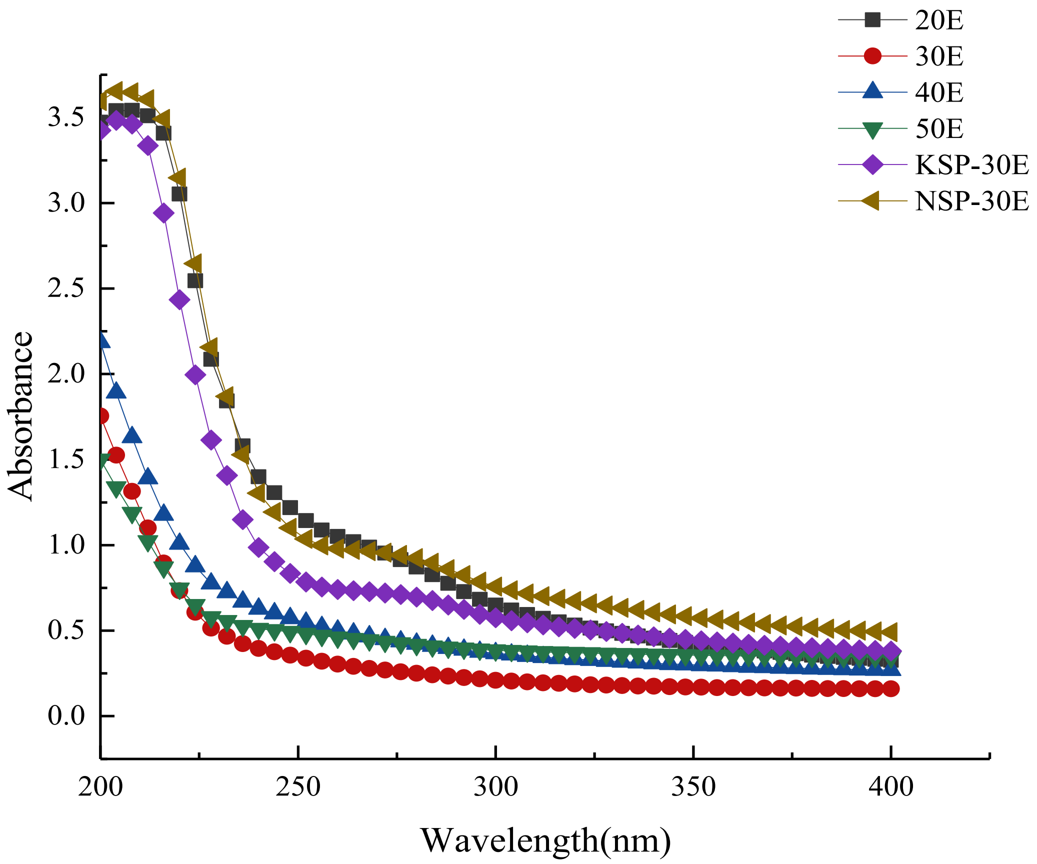

Figure S1. UV wavelength scanning spectra of polysaccharide fractions. E: The polysaccharide fractions of 20E, 30E, 40E and 50E were obtained by sequentially adding ethanol to the different concentrations (20%–50%) in water extract of superfine grinding assisted extraction (SP). NSP: sodium hydroxide extract, KSP: potassium hydroxide extract.
